# Pregnancy-Induced Thrombotic Thrombocytopenic Purpura Complicated by Atypical Posterior Reversible Encephalopathy Syndrome and Acute Pancreatitis: A Rare Presentation

**DOI:** 10.7759/cureus.90482

**Published:** 2025-08-19

**Authors:** Madhu Kiran Reddy Turupu, Nikhil Kumar Balagoni, Rithik Naik Korra, Uma Shailendri Rayudu, Neha Bijjigum, Jaswanth Soorisetty, Vishal Loney, Praveen Bharath Saravanan

**Affiliations:** 1 General Medicine, Osmania Medical College, Hyderabad, IND; 2 Internal Medicine, Osmania Medical College, Hyderabad, IND; 3 Internal Medicine, Gitam Institute of Medical Sciences and Research, Visakhapatnam, IND; 4 Internal Medicine, Kakatiya Medical College, Warangal, IND; 5 Internal Medicine, Saint James School of Medicine, Arnos Vale, VCT; 6 Internal Medicine, K.A.P. Viswanatham Government Medical College, Tiruchirappalli, IND

**Keywords:** acute kidney injury, acute pancreatitis, plasma exchange, posterior reversible encephalopathy syndrome, pregnancy, thrombotic thrombocytopenic purpura

## Abstract

Pregnancy is a documented trigger for thrombotic thrombocytopenic purpura (TTP), a rapidly progressing condition that is often misdiagnosed as HELLP (hemolysis, elevated liver enzymes, low platelets) syndrome. Here, we present the case of a 28-year-old pregnant female, gravida 2 para 1, who was admitted for complaints of seizures, vomiting, fever, decreased perception of fetal movements, and visual impairment. She was found to have posterior reversible encephalopathy syndrome, acute kidney injury, and acute pancreatitis, along with her diagnosis of TTP. She improved with plasma exchange and recovered well. Maternal and fetal survival in TTP patients depends heavily on early diagnosis, swift initiation of therapeutic plasma exchange, and vigilant monitoring throughout pregnancy and the postpartum period. This case emphasizes the importance of ruling out differentials often grouped with TTP in resource-limited settings.

## Introduction

Thrombotic thrombocytopenic purpura (TTP) is a microangiopathic hemolytic anemia that leads to blood clots in small blood vessels. TTP is characterized by fever, hemolytic anemia, thrombocytopenia, and renal and neurologic dysfunction. It results from a congenital or acquired absence or decrease of the von Willebrand factor (vWF)-cleaving protease ADAMTS13 (a disintegrin and metalloproteinase with a thrombospondin type 1 motif member 13) [1]. Decreased ADAMTS13 activity causes microthrombi formation, leading to end-organ ischemia and damage. Although ADAMTS-13 deficiency is necessary to cause TTP, it is usually insufficient to induce this clinical syndrome.

TTP can present at any age but most commonly in pregnant females [2,3]. Pregnancy combined with TTP is a rapidly progressing condition that is often misdiagnosed as an obstetric condition such as preeclampsia or HELLP (hemolysis, elevated liver enzymes, low platelets) syndrome [4]. Here, we present the case of a pregnant 28-year-old female who was admitted for complaints of seizures, vomiting, and fever following spontaneous delivery.

## Case presentation

A previously healthy 28-year-old female, gravida 2, para 1, was referred to our tertiary care hospital with complaints of seizures, fever, and vomiting that developed immediately after spontaneous delivery due to intrauterine fetal death at a primary healthcare center the day following delivery. The patient was on regular antenatal care, with no comorbidities, and was only on prenatal vitamins, iron-folic acid, and calcium supplements, and not on any other medications. Her previous pregnancy was uneventful.

Initial laboratory results revealed a hemoglobin level of 13.5 g/dL, a white blood cell (WBC) count of 25,000/µL, a platelet count of 49,000/µL, and a serum creatinine level of 2.49 mg/dL. Subsequently, the patient developed a headache followed by decreased urine output and blurring of vision. On examination, she exhibited mild icterus, bilateral pitting pedal edema, and visual impairment (able to perceive hand movements only), with no motor, sensory, or cerebellar involvement. The patient’s blood pressure was 150/90 mmHg. We initially considered HELLP syndrome, but liver enzymes were not markedly increased, making it less likely; hence, we considered other differentials such as eclampsia, acute kidney injury (AKI), posterior reversible encephalopathy syndrome (PRES), and cerebral sinus venous thrombosis. Consequent blood work revealed increased aspartate aminotransferase/alanine aminotransferase and lactate dehydrogenase, as well as worsening thrombocytopenia. A peripheral smear was then ordered, revealing schistocytes, anisocytosis, and microcytes, suggesting hemolysis. Coombs was negative, ruling out immune-mediated hemolysis. A corticosteroid was initiated, and due to worsening renal function, the patient was placed on dialysis. Investigations were ordered and monitored throughout the course of hospitalization (Table 1).

**Table 1:** Laboratory results during the course of hospitalization. WBC = white blood cells; Hb = hemoglobin; AST = aspartate aminotransferase; ALT = alanine aminotransferase

Day	WBC (mm^3^)	Hb (g/dL)	Platelets (mm^3^)	Urea (mg/dL)	Creatinine (mg/dL)	Total Bilirubin (mg/dL)	Direct Bilirubin (mg/dL)	AST (U/L)	ALT (U/L)	Amylase (U/L)	Lipase (U/L)
Day 1	25,000	13.5	49,000	-	2.49	2.35	0.6	-	-	-	-
Day 3	32,900	10.8	45,000	68	4.9	3.4	0.8	279	58	-	-
Day 4	30,660	10.0	55,000	71.9	5.69	3.0	0.7	50	47	770	1,930
Day 6	23,820	9.9	82,000	95.6	7.49	2.82	0.6	48	86	440	1,003
Day 8	13,210	7.6	64,000	-	-	-	-	36	50	-	-
Day 9	11,540	6.9	63,000	71.7	5.96	2.8	1.0	43	17	428	922
Day 10	-	-	-	53.3	5.43	-	-	-	-	-	-
Day 11	-	-	-	42	4.7	2.7	0.9	39	12	-	-
Day 12	8,430	6.7	86,000	60	6.53	2.3	0.4	37	8.32	283	476
Day 13	10,540	7.8	130,000	51	4.85	-	-	-	-	-	-
Day 16	10,450	8.0	100,000	55.8	4.8	-	-	-	-	-	-
Day 18	5,000	8.1	93,000	74	5.76	-	-	-	-	-	-
Day 20	4,200	7.8	81,000	67	5.87	-	-	-	-	74	176

CT scan of the brain revealed subcortical white matter edema in the parietal and occipital lobes, consistent with PRES. Three days after admission, the patient complained of severe epigastric abdominal pain. A CT scan of the abdomen showed a bulky pancreas, along with bilateral non-obstructive renal calculi. Serum markers such as serum amylase (770 U/L) and lipase (1,930 U/L) were raised, indicating acute pancreatitis. Supportive care for the same was initiated. The patient was continued on hemodialysis and corticosteroid therapy. Subsequent neurological assessments showed improvement in her visual acuity, with the patient able to count fingers, indicating a positive response to treatment for PRES.

**Figure 1:**
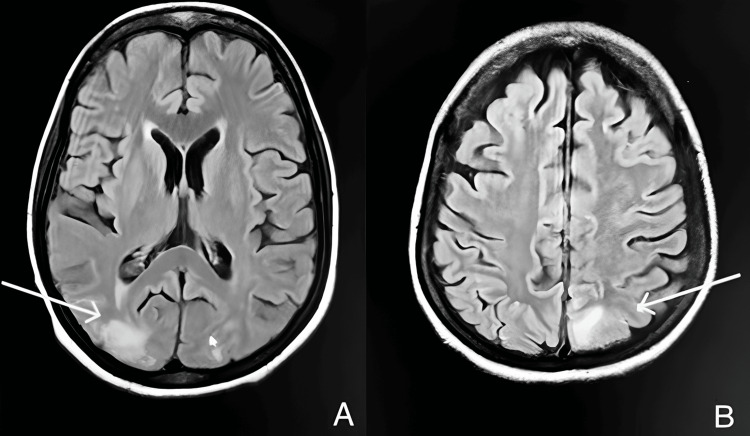
MRI findings consistent with atypical posterior reversible encephalopathy syndrome. (A) Axial T2-weighted and (B) fluid-attenuated inversion recovery MRI showing hyperintensities involving the cortical and subcortical white matter of the bilateral parieto-occipital regions. These findings are consistent with atypical posterior reversible encephalopathy syndrome.

An MRI of the brain confirmed bilateral symmetrical hyperintensities in the parieto-occipital regions, consistent with atypical PRES (Figure 1). Worsening thrombocytopenia and creatinine made HELLP unlikely, and a kidney biopsy was performed, revealing near-diffuse cortical necrosis with focal changes suggestive of underlying thrombotic microangiopathy. Complement levels (C3 and C4) were normal, and workup for other thrombotic microangiopathies (TMAs) was initiated. ADAMTS13 autoantibody levels were found to be markedly increased at 48.0 AU/mL, confirming the diagnosis of TTP.

Nineteen days into hospitalization, serum amylase and lipase levels decreased, indicating a positive response to supportive care for pancreatitis. Plasma exchange therapy was initiated, showing clinical improvement. Due to the complexity of overlapping symptoms, including PRES, AKI, and acute pancreatitis, early diagnosis and initiation of plasma exchange were delayed. Due to worsening creatinine levels from day two, she was on continued hemodialysis and steroids until discharge and was found to have no complications on post-hospitalization follow-up in the following months.

## Discussion

TTP is a multisystem disorder marked by microangiopathic hemolytic anemia, thrombocytopenia, neurological abnormalities, and renal involvement. Pregnancy is known to create an environment favoring coagulability through increased procoagulant factors, reduced fibrinolysis, loss of endothelial cell thrombomodulin, and decreased ADAMTS-13 activity, which, as a consequence, triggers acute TTP episodes [3]. TTP occurs in about 2 out of every 100,000 pregnancies [5]. Nearly half of acute TTP cases occur in reproductive-age women, with pregnancy-related TTP accounting for 12% to 25% of adult-onset cases [2]. The majority of these cases present during delivery or immediately postpartum, posing greater risks to both mother and fetus if left untreated [6]. Fetal death is typically due to placental infarction, while maternal mortality can arise from acute renal failure, disseminated intravascular coagulation, or severe thrombocytopenia [4].

Differentiating TTP from other TMAs related to pregnancy, such as HELLP syndrome, preeclampsia, systemic lupus erythematosus, antiphospholipid syndrome, and acute fatty liver of pregnancy (AFLP), is challenging due to overlapping clinical presentations [7]. TTP is primarily caused by a congenital or an immune-mediated acquired deficiency of the vWF-cleaving protease ADAMTS-13, leading to the formation of microthrombi, end-organ ischemia, and damage [1]. The organs often affected include the heart, brain, kidneys, and adrenal glands, with the pancreas also potentially involved [8].

Our patient presented with headaches, tonic-clonic seizures, and blurred vision [9]. Headaches and seizures are more common presentations compared to blurred vision. Brain imaging (CT and MRI) revealed cerebral edema in the posterior regions, with parieto-occipital white matter changes indicative of PRES [10,11]. PRES is characterized by symmetrical, posterior subcortical vasogenic edema. It is a rare but known complication in pregnancy, often linked with hypertensive disorders [12,13]. Our patient did not exhibit hypertension. The authors point out that PRES may also result from alternative mechanisms, such as endothelial dysfunction or immune-mediated responses [14].

In severe preeclampsia, PRES is associated with endothelial disruption and cerebral edema. However, its pathophysiology remains unclear in patients without preeclampsia [13]. A study by Burrus et al. [13] suggests that kidney injury is associated with PRES in hospitalized TTP patients [14]. Our patient developed AKI, necessitating continuous hemodialysis. A kidney biopsy confirmed TMA, aiding in diagnosis and ruling out other causes of kidney failure [15]. We believe, in similar cases, early diagnosis is advisable to differentiate TMA causes and prevent severe complications such as end-stage renal disease, myocardial ischemia, seizures, strokes, or even death [16].

During hemodialysis for AKI, the patient experienced abdominal pain, and an ultrasound confirmed acute pancreatitis, supported by raised serum markers. The microvascular thrombi in TTP can compromise pancreatic blood flow, leading to ischemia and progressing to acute pancreatitis [17]. Although pancreatitis is considered a consequence of TTP, some studies suggest it might trigger TTP in certain cases, emphasizing the complexity of this correlation [18,19]. In our patient, TTP and acute pancreatitis were diagnosed simultaneously. Given the rarity of pancreatitis in pregnancy without underlying biliary disease, it is plausible that TTP was the primary cause of pancreatitis here [20,21]. This case emphasizes the need to consider pancreatitis in TTP patients with abdominal pain and evaluate TTP in acute pancreatitis patients with thrombocytopenia. The patient’s condition improved with aggressive plasma exchange therapy.

The mainstay treatment of TTP is plasma exchange, which involves replacing 1 to 1.5 plasma volumes with fresh frozen plasma and administering corticosteroids (prednisone at 1 mg/kg/day). Plasma exchange effectively removes autoantibodies and ultra-large vWF multimers while replenishing functional ADAMTS-13 and fresh plasma components [22]. Accurate and timely diagnosis in pregnancy is critical because TTP can be almost uniformly fatal without prompt, targeted therapy. Unlike conditions such as HELLP syndrome, where delivery of the fetus is the definitive treatment, TTP is unaffected by uterine evacuation, and platelet transfusion is contraindicated as it may exacerbate the condition [23]. In contrast, the role of plasma exchange therapy remains controversial for syndromes such as preeclampsia and AFLP [7].

However, caplacizumab, a monoclonal antibody that blocks the adhesion of vWF multimers to platelets, was reported to be used in two pregnant individuals with refractory immune-mediated TTP [24,25]. In both cases, caplacizumab effectively improved platelet counts in refractory disease, though adverse pregnancy outcomes could not be prevented. In the first case, the patient developed preeclampsia, severe growth restriction, oligohydramnios, and placental hydrops, leading to termination of pregnancy [24]. In the second case, preterm delivery was required due to fetal distress, but the neonate recovered well [25]. Given that these are the only two cases reported in the literature so far, usage of this drug should be based on shared decision-making and should involve careful risk-benefit discussion with the patient. It has also been proposed that caplacizumab may theoretically increase the risk of maternal and neonatal bleeding; therefore, its use should be reserved for refractory cases [25].

## Conclusions

Routine antenatal screening, such as blood pressure and urine protein, can help prevent pregnancy-related complications such as hypertension. In patients with thrombocytopenia associated with neurological and renal manifestations, TTP should be suspected, and maternal and fetal outcomes are dependent on prompt initiation of plasma exchange therapy, along with close monitoring of platelet counts and serum lactate dehydrogenase levels. Early ADAMTS13 testing should be prioritized in suspected cases. A multidisciplinary approach involving obstetricians, hematologists, and nephrologists is essential for the successful management of TTP during pregnancy to ensure comprehensive care.
